# Influence of Laser Power on CoCrFeNiMo High-Entropy Alloy Coating Microstructure and Properties

**DOI:** 10.3390/ma18112650

**Published:** 2025-06-05

**Authors:** Shuai Li, Fuheng Nie, Jiyuan Ding, Guijun Mao, Yang Guo, Tianlan Cao, Chong Xiang, Honggang Dong

**Affiliations:** 1 State Key Laboratory of Clean and Efficient Turbomachinery Power Equipment, Deyang 618000, China; maoguijun0211@163.com (G.M.); guoyang0192@163.com (Y.G.); caotianlan333@163.com (T.C.); xiangchong0311@163.com (C.X.); 2 School of Mechanical Engineering, North China University of Water Resources and Electric Power, Zhengzhou 450045, China; dingjiyuan65959610@163.com; 3 Dongfang Electric Corporation Dongfang Turbine Co., Ltd., Deyang 618000, China; 4 School of Materials Science and Engineering, Dalian University of Technology, Dalian 116024, China; donghg@dlut.edu.cn

**Keywords:** high-entropy alloy coatings, coaxial powder-feeding laser cladding, CoCrFeNiMo, laser power

## Abstract

This work studies the fabrication of CoCrFeNiMo high-entropy alloy (HEA) coatings via coaxial powder-fed laser cladding, addressing porosity and impurity issues in conventional methods. The HEA coatings exhibited eutectic/hypereutectic microstructures under all laser power conditions. A systematic investigation of laser power effects (1750–2500 W) reveals that 2250 W optimizes microstructure and performance, yielding a dual-phase structure with FCC matrix and dispersed σ phases (Fe-Cr/Mo-rich). The coating achieves exceptional hardness (738.3 HV_0.2_, 3.8× substrate), ultralow wear rate (4.55 × 10^−5^ mm^3^/N·m), and minimized corrosion current (2.31 × 10^−4^ A/cm^2^) in 3.5 wt.% NaCl. The friction mechanism of the CoCrFeNiMo HEA coating is that in high-speed friction and wear, the oxide film is formed on the surface of the coating, and then the rupture of the oxide film leads to adhesive wear and abrasive wear. The corrosion mechanism is the galvanic corrosion caused by the potential difference between the FCC phase and the σ phase.

## 1. Introduction

High-entropy alloys (HEAs), an emerging category of metallic materials, are characterized by their unique composition; they contain five or more principal elements blended in approximately equal molar proportions. The highly disordered atomic arrangement induces a transition from ordered to random atomic distribution, resulting in four characteristic effects: thermodynamic high-entropy effect, kinetic sluggish diffusion effect, structural lattice distortion effect, and performance-related “cocktail effect” [1]. As an emerging alloy system, HEAs have demonstrated exceptional properties and promising potential in coating applications [2], positioning them at the forefront of global materials research.

**Figure 1:**
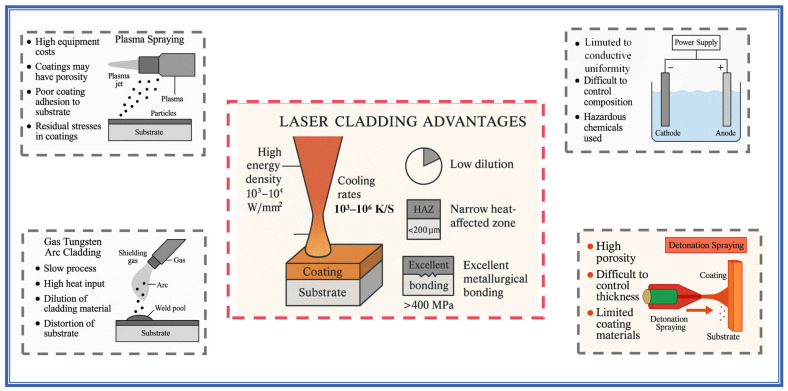
The advantages of laser cladding.

HEA coating technologies have emerged as a research focus in materials science due to their exceptional properties, with multiple fabrication processes being developed to date [3]. Based on distinct processing principles, these techniques can be primarily categorized into three groups: 1. deposition methods (e.g., electrochemical deposition [4] and physical vapor deposition (PVD [5])); 2. spraying techniques (including HVOF spraying [6], cold spraying [7], and plasma spraying processes [8,9]); and 3. cladding techniques (encompassing plasma cladding [10,11], gas tungsten arc cladding [12], electron beam cladding [13], and laser cladding [14,15,16]). As shown in Figure 1, among these, laser cladding has demonstrated unique advantages through its high energy density (typically 10^3^–10^4^ W/mm^2^) and rapid cooling rates (10^3^–10^6^ K/s), enabling the fabrication of coatings with superior characteristics: low dilution rates, narrow heat-affected zones (HAZ < 200 μm), and excellent metallurgical bonding (interface strength > 400 MPa) [17]. These attributes have rendered it particularly advantageous for critical applications in marine engineering and precision machinery manufacturing.

Current research in laser cladding coating fabrication predominantly employs a two-stage methodology: initial powder preplacement onto the substrate surface, followed by laser irradiation to achieve simultaneous fusion of the deposited material with the base substrate, thereby forming high-quality coatings. Zhang et al. [18] synthesized CoCrFeNiTi HEA coatings on 304SS via laser cladding using ball-milled pre-alloyed powders. The coating microstructure comprises an FCC solid-solution matrix and Fe-Cr-enriched Laves phase precipitates, accompanied by directionally solidified columnar grains and eutectic morphology. Dual strengthening mechanisms (solid-solution and second-phase reinforcement) achieved 2.4 times the substrate hardness (568 HV_0.2_) and a lower erosion wear rate, demonstrating exceptional wear resistance. Zeng et al. [19] prepared a laser-clad coating on cleaned Ti-6Al-4V substrates by pre-depositing a 2 mm layer of ball-milled blended Al/Co/Cr/Fe/Ni/Ti powders. The coating exhibited a dual-phase microstructure: BCC phases predominantly located in dendritic cores and FCC phases concentrated in interdendritic regions. Oxidation tests revealed exceptional performance with a parabolic rate constant tenfold lower than that of the substrate. High-temperature tribological evaluation at 600 °C demonstrated a wear volume loss of 4.97 × 10^−4^ mm^3^/N·m, representing a 13-fold reduction compared to the substrate. Zhang et al. [20] fabricated laser-clad coatings on Q235 steel using Fe/Co/Ni/Cr/Cu pre-mixed powders with individual additions of trace Si, Mn, and Mo elements. The coatings maintained a single FCC structure, while elemental doping significantly enhanced microhardness, elevated softening onset temperature, and reduced corrosion current density through synergistic solid-solution strengthening and passive film optimization. Currently, most research on laser cladding HEA coatings focuses on pre-coating the substrate with powder, followed by scanning with a high-energy laser beam to form the coating, but this method relies on the use of organic solvents. This can lead to the formation of pores and the introduction of impurities during the cooling process of the laser melt pool. In contrast, coaxial powder-fed laser cladding technology is suitable for surfaces with complex geometries and allows for precise control of the cladding area without the need for binders. This eliminates issues related to porosity and impurities while offering broader application potential.

The transition metal elements Co, Cr, Fe, and Ni are conventionally selected as primary constituents of HEAs due to their comparable atomic radii. However, FeCoCrNi HEA coatings predominantly form single-phase FCC structures, whose low hardness limits their applicability in high-stress environments [21]. To address this, researchers have opted to introduce additional principal elements to engineer phase-structured HEAs with distinct physicochemical properties. For instance, Al additions induce dual-phase solid solution microstructures via FCC to BCC phase transformation mechanisms [22]. Nevertheless, single solid-solution HEAs still demonstrate suboptimal performance under complex tribological and corrosive service conditions.

Mo has gained prominence for its capacity to form intermetallic compounds with exceptional physical properties, mechanical robustness, and thermal stability. Incorporating Mo into quaternary FeCoCrNi HEAs promotes the precipitation of topologically close-packed (TCP) phases (e.g., σ phase), which generate residual compressive stresses to inhibit crack propagation and enhance fatigue resistance [23]. The synergistic coexistence of FCC matrices and hard intermetallic compounds endows these coatings with superior comprehensive mechanical properties, enabling exceptional performance in multifactorial service environments involving wear–corrosion interactions [24]. Chang et al. [25] mechanically mixed different proportions of Co, Cr, Fe, Ni, and Mo powders to obtain CoCrFeNiMo*_x_* (*x* = 0, 0.5, 1.0, 1.5) mixed powders. After blending the powders with an organic solvent, they were pre-coated on the surface of AISI1045 steel to prepare laser cladding HEA coatings. The CoCrFeNiMo*_x_* coatings are primarily composed of an FCC phase. The addition of Mo increases the lattice distortion of the FCC matrix. When the Mo content is high (*x* ≥ 1.0), a small amount of BCC phase or σ phase appears, and the microstructure transitions from columnar grains to equiaxed grains. At higher Mo contents, grain refinement occurs. CoCrFeNiMo_1.5_ demonstrates superior hardness and exceptional wear resistance, primarily attributed to three synergistic mechanisms: solid-solution strengthening, refined grain structures, and precipitation hardening induced by the σ phase enriched with Fe, Cr, and Mo. Zeng et al. [26] used ball-milled Co, Cr, Fe, Ni, Mo, Ti, and Mn powders to prepare coatings on the surface of low-carbon steel through laser cladding. The CoCrFeNi and CoCrFeNiMn HEA coatings exhibit single-phase FCC structures, while the CoCrFeNiTi coating comprises FCC, Laves, and η phases, and the CoCrFeNiMo coating consists of FCC and σ phases. Among these, the CoCrFeNiMo coating demonstrates the highest hardness (~700 HV) and optimal wear resistance due to σ-phase strengthening, with oxidative wear identified as the dominant wear mechanism. However, the multiphase structures induce galvanic corrosion effects, resulting in comparatively inferior corrosion resistance for both CoCrFeNiMo and CoCrFeNiTi coatings.

Among various HEA systems, the CoCrFeNiMo series has been particularly noteworthy for its dual-phase structure and has proven to be a high-performance material in diverse fields, including marine engineering, aerospace, and high-speed rail applications [27]. Currently, there has been no research on the preparation of CoCrFeNiMo HEA coatings using coaxial powder-fed laser cladding, nor on the effect of laser cladding parameters on the performance of the coatings. In the process of fabricating CoCrFeNiMo HEA coatings via coaxial powder-feeding laser cladding, laser power plays a critical regulatory role in the microstructural evolution and macroscopic performance of the coatings. Notably, while existing studies predominantly focus on multi-parameter coupling effects, there is still a gap in the systematic study of the coating structure evolution, tribological properties, and electrochemical corrosion mechanisms under univariate regulation strategies (such as laser power gradient changes). This study employed coaxial powder-feeding laser cladding to deposit CoCrFeNiMo coatings on Q355 steel substrates. By systematically varying four laser power parameters, this study elucidated the influence of laser energy density on microstructure and properties.

## 2. Materials and Methods

### 2.1. Coating Deposition

**Figure 2:**
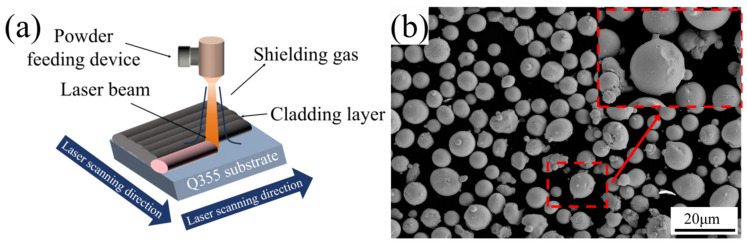
(**a**) Schematic illustration of the laser cladding in this study; (**b**) SEM morphologies of CoCrFeNiMo HEA powders.

The experimental platform employed Q355 steel substrates (100 mm × 100 mm × 20 mm). The Q355 steel used complies with ATSM A992 [28], and the quantitative characterization of its elemental composition is shown in Table 1. As the cladding material, CoCrFeNiMo HEA powder was procured from Avimetal Powder Metallurgy Technology Co., Ltd. (Beijing, China). The physical appearance of the HEA powder is depicted in Figure 2b.

**Figure 3:**
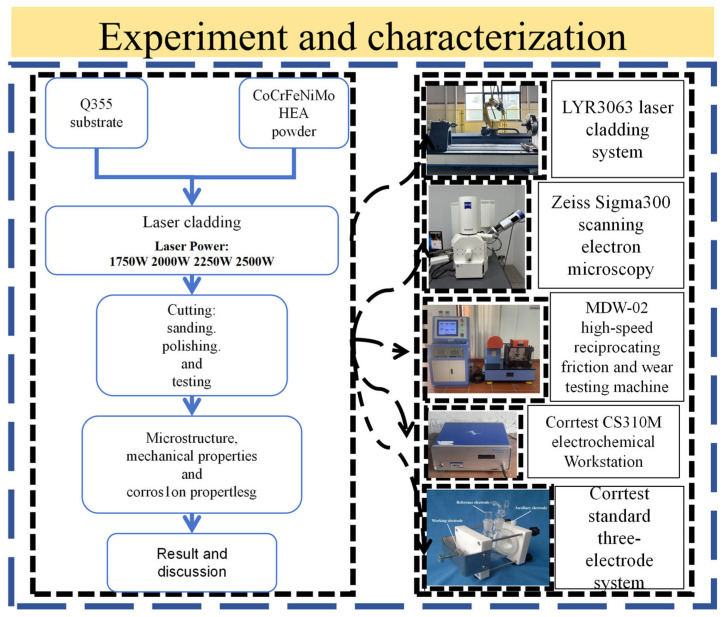
Experimental equipment and procedures.

As shown in Figure 3, laser deposition was executed via a LYR3063 system (LYR3063, Jinan Senfeng Technology Co., Ltd., Jinan, China) under four distinct power configurations: 1750, 2000, 2250, and 2500 W. Other process parameters were standardized as follows: 150 cm/min scanning velocity, 1.5 rpm powder feed rate, 2 mm beam diameter, and 50% overlap ratio with 1 mm/pass advancement.

### 2.2. Characterization of the Coatings

In this study, the coatings were first polished step-by-step. Sandpapers of grit sizes 300, 500, 1000, 1200, 1500, and 2000 were used in sequence for initial polishing. Subsequently, W1.5 diamond paste was employed to create a mirror-smooth surface on the coatings. After that, the polished samples underwent a 30 s etching process in a mixed acid solution with a HCl: HNO_3_ volume ratio of 3:1.

Multi-scale characterization of the coatings was conducted employing a systematic analytical protocol: phase identification via X-ray diffraction (XRD, X’Pert PRO MPD, PANalytical B.V., Almelo, The Netherlands) with Cu Kα radiation (λ = 1.5406 Å) at 40 kV/40 mA, operating in 2θ geometry with 5°/min scanning resolution; metallographic examination using optical microscopy (OM, GX53, Olympus Corporation, Tokyo, Japan); and coupled with scanning electron microscopy (SEM, Sigma 300, Carl Zeiss AG, Oberkochen, Germany) operated in secondary electron imaging mode and back-scattered electron imaging (15 kV accelerating voltage) integrated with energy-dispersive X-ray spectroscopy (EDS, OXFORD Xplore 30, OXFORD INSTRUMENTS, London, UK) for elemental microanalysis.

Microhardness characterization was systematically conducted using a Vickers microhardness testing system (HVS-1000, Shanghai Precision Instruments Co., Ltd., Shanghai, China) under standardized indentation protocol (200 g load, 15 s dwell time) following strict compliance with ASTM E384-17 [29]. An average of 100 points were taken within a 3 mm × 3 mm square area on the surface to create a surface hardness contour map. Cross-sectional hardness profiling was performed along the coating–substrate interface normal direction using metallographically prepared cross-sections, with automated stage positioning at 0.1 mm incremental steps from the coating surface to the substrate interface, capturing the hardness gradient evolution across the coating thickness.

Tribological performance evaluation was conducted using the reciprocating high-speed friction and wear tester (MDW-02, Jinan Yihua Tribology Testing Technology Co., Ltd., Jinan, China) under dry sliding conditions, according to standard ASTM G133-05 [30]. The test protocol employed a 6 mm reciprocating stroke with 20 N load at 10 Hz oscillation frequency (equivalent to 0.12 m/s sliding velocity), generating a total sliding distance of 216 m over a 30 min test duration. Post-test wear track characterization was performed using white-light interferometry (Sneox 090, QUESTAR, St. Miami, FL, USA) to quantify three-dimensional wear volume. Wear mechanisms were analyzed through the SEM-EDS examination of wear track cross-sections.

Electrochemical corrosion behavior was quantitatively evaluated through a sequential testing protocol per ASTM G3-14 [31]. The electrochemical analysis system (CS310M, Wuhan Corrtest Instrument Co., Ltd., Wuhan, China) was configured with a conventional three-electrode array: platinum mesh counter electrode, saturated calomel reference electrode (SCE), and working electrode (coating specimen with 0.38 mm^2^ geometric surface area). Open-circuit monitoring (OCP) stabilization over 3600 s was performed until potential drift < 2 mV/min. Otentiodynamic polarization: voltage scanning from −1.0 V to +0.5 V vs. OCP at a 10 mV s^−1^ scan rate, with Tafel extrapolation for corrosion current density determination.

## 3. Results

### 3.1. Phase Composition and Microstructure

**Figure 4:**
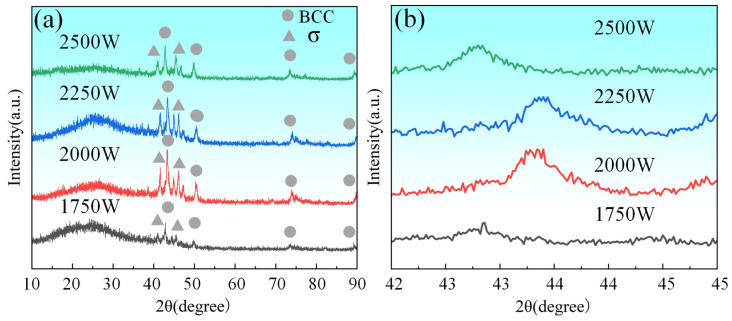
XRD patterns of the FeNiCoCrMo HEA coating with different laser powers: (**a**) overall view (**b**) enlarged view from a specific angle.

According to the XRD analysis results presented in Figure 4, the CoCrFeNiMo HEA coating exhibited a coexistence of FCC and σ phases, with the FCC phase being the dominant constituent. The FCC phase was primarily composed of Ni, Cr, Co, and Mo. The σ phase, characterized as a topologically close-packed structure, displayed two distinct compositional characteristics: one predominantly consisting of Fe and Cr, and the other mainly comprising Fe and Mo [32,33].

Furthermore, as evident from Figure 4b, the diffraction peak intensity initially increased and subsequently decreased with increasing laser power. Simultaneously, the diffraction peak positions initially shifted noticeably to the right and then to the left. These variations likely reflect alterations in the lattice parameters and dynamic adjustments in the stress state within the coating. The observed peak shifts are also correlated with changes in interatomic spacing in the coating [34].

As reported in Reference [32], for Mo*_x_*CoCrFeNi HEA, the formation of additional Mo-containing phases beyond the FCC phase was observed when the molar fraction of Mo exceeded 0.3. This phenomenon occurs because in HEA systems composed of Fe, Ni, Co, Cr, and Mo, higher Mo content tends to suppress FCC phase formation. In this study, Q355 steel was employed as the substrate, with Fe being its primary constituent. With increasing laser power, more Fe from the substrate was incorporated into the coating. This led to an elevated Fe content in the coating, which subsequently promoted the formation of Fe- and Mo-containing σ phases, thereby reducing the diffraction intensity of the FCC phase.

The FCC phase represents a typical solid solution structure, whereas the σ phase belongs to the topologically close-packed (TCP) phase category. In HEA systems, TCP phases tend to form when the electro-negativity difference (Δ*X*) exceeds 0.133. The Δ*X* can be calculated using the following Equation (1) [35]:

(1) $$∆X=\sqrt{\int_{i=1}^{N}{C_{i}\left(X_{i}-\bar{x}\right)}^{2}}$$

Through calculation, the Δ*X* of the CrCoFeNiMo HEA was determined to be 0.1612. Compared with the single FCC phase observed in the CrCoFeNi HEA, the CrCoFeNiMo HEA coatings exhibited a dual-phase structure consisting of FCC and σ phases.

When Mo is added to the CoCrFeNi HEA, it first dissolves into the FCC lattice. Due to Mo’s large atomic radius, it causes severe lattice distortion in the FCC phase. When the solubility limit of Mo in the FCC phase is reached, Mo-rich σ phase precipitates within the FCC matrix. During the solidification of the CoCrFeNiMo HEA coating, the σ phase exhibits different morphologies depending on the temperature at which it precipitates during the eutectic reaction. Under higher laser power conditions, Mo elements were sufficiently melted and formed alloy compounds with other constituent elements, inducing lattice distortion in the FCC phase. This distortion manifested as a rightward shift in the diffraction peaks. Additionally, the increase in laser power caused a higher dilution rate of the coating, particularly due to the enhanced incorporation of Fe from the Q355 substrate into the coating, thereby further elevating the Fe content in the coating. The increased laser power also improved the fluidity of the molten pool, facilitating better mixing between different phases. However, excessively high laser power led to excessive participation of Fe in the formation of the HEA, which might cause a leftward shift in the diffraction peaks, reflecting further alterations in lattice parameters.

**Figure 5:**
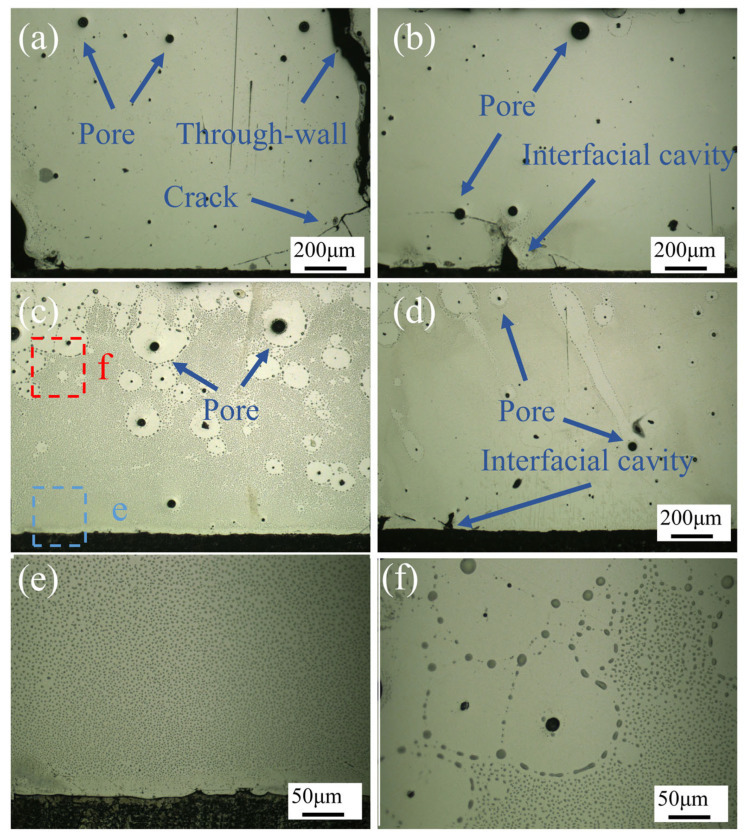
OM images of CoCrFeNiMo coatings with different laser powers: (**a**) 1750 W; (**b**) 2000 W; (**c**) 2250 W; (**d**) 2500 W. (**e**,**f**) present magnified views of the corresponding regions in (**c**).

Figure 5 illustrates OM images of the CoCrFeNiMo HEA coating, corresponding to different laser power settings: 1750 W, 2000 W, 2250 W, and 2500 W. The images clearly revealed the presence of pores, through-thickness cracks, and interfacial cavities in the coatings produced at 1750 W, 2000 W, and 2500 W.

At the laser power of 1750 W, large pores were observed at the top surface of the coating, while fine pores and transverse cracks were present at the coating–substrate interface. Additionally, through-thickness cracks were identified, resulting in poor forming quality. When the power was increased to 2000 W, pores remained in the coating, but the number of cracks significantly decreased, with through-thickness cracks disappearing. However, interfacial cavities emerged at the coating–substrate interface. At 2250 W, coating defects were substantially reduced, with only pores being observed, demonstrating significantly improved forming quality compared to the other three parameters. However, at the highest power of 2500 W, coating defects increased again, exhibiting not only pores but also pronounced interfacial cavities.

During the laser heating and cooling process, the powder and substrate were melted by the high-energy laser beam. Upon cooling, the cladding track solidified, and the substrate formed a large molten pool. The sides and bottom of the molten pool cooled more rapidly than the central region. When the center solidified, this difference was compensated by plastic deformation in the center. Additionally, during multi-track cladding, the maximum stress increased with the deposition of adjacent tracks. The stress mainly consisted of three components: first, the volume shrinkage from liquid to solid phase during the solidification of the HEA powder; second, the microstructural stress caused by volume contraction as the solid phase cooled to room temperature; and third, the martensitic phase transformation induced by the thermal effect on the substrate surface. These combined effects led to crack formation in the coating. Farahmand et al. [36] obtained similar results through simulation, showing significant residual tensile stress concentration within the composite coating, residual compressive stress in the heat-affected zone, and varying residual stress levels between cladding tracks, with lower residual stress at earlier cladding positions. Q355 steel undergoes a martensitic transformation near 400 °C. At a laser power of 2500 W, excessive heat input intensified the volume expansion caused by this transformation. In Wang’s study [37], the martensitic transformation of the substrate increased the tensile stress in the coating and was identified as a primary cause for the formation of cavities at the bottom of the coating.

At lower laser power settings, insufficient dilution between the coating and substrate prevented the formation of effective metallurgical bonding, resulting in inadequate interfacial strength. With increasing laser power, enhanced dilution between the coating and substrate promoted stronger metallurgical bonding, significantly reducing coating defects. Nevertheless, when the laser power was further increased to 2500 W, excessive heat input not only induced martensitic transformation in the substrate but also generated substantial interfacial cavities due to the mismatch in thermal expansion coefficients between the coating and substrate [38].

Figure 5e,f present magnified views of the corresponding regions in c, revealing that the CoCrFeNiMo HEA coating primarily consists of two distinct phases: a yellow phase and a black phase. In this context, the yellow phase represents the matrix, while the black phase, serving as the secondary phase, is dispersed throughout the matrix with a noticeably increasing distribution density from bottom to top. Notably, the black phase exhibits a disappearance phenomenon around the pores. Based on previous XRD analysis results, we can infer that the yellow phase corresponds to the FCC-structured matrix phase, whereas the black phase represents the σ phase, which is an enhanced phase formed through specific elemental enrichment.

**Figure 6:**
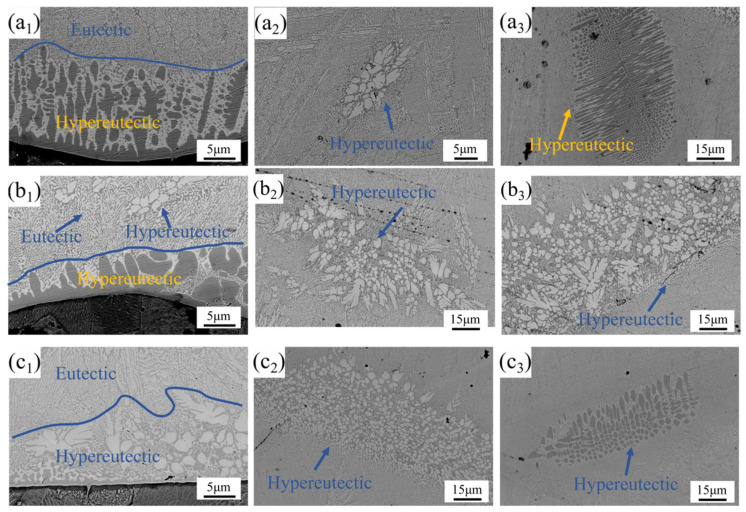
SEM images of CoCrFeNiMo coatings with different laser power: (**a_1_**–**a_3_**) 2000 W; (**b_1_**–**b_3_**) 2250 W; (**c_1_**–**c_3_**) 2500 W.

Figure 6 displays the SEM images obtained under different laser power settings. Due to the poor forming quality of the coating at 1750 W, this investigation focused on three representative conditions: 2000 W (Figure 6(a_1_–a_3_)), 2250 W (Figure 6(b_1_–b_3_)), and 2500 W (Figure 6(c_1_–c_3_)). These images reveal a complex tri-phase microstructure comprising gray, white, and black phases. Specifically, the interfacial regions near the coating–substrate boundary (shown in Figure 6(a_1_,b_1_,c_1_)) exhibited coarse-grained structures. The grain morphology demonstrated significant evolution with increasing laser power: gradual refinement of grain size and progressive color transition from black to gray.

As shown in Figure 6(a_1_,b_1_,c_1_), the coating’s interfacial region exhibited a tri-phase (black/gray/white) microstructure comprising eutectic and hypereutectic structures. At 2000 W laser power, the near-interface zone predominantly displayed hypereutectic structures that gradually transitioned to eutectic morphology. When the power increased to 2250 W, the hypereutectic area fraction decreased, and the structure above the large grain region evolved into a eutectic–hypereutectic composite structure. Further increasing the power to 2500 W resulted in the complete disappearance of the black and a full transition from a hypereutectic structure to a eutectic structure.

The mid and upper regions of the coating (as shown in Figure 6(a_2_,a_3_,b_2_,b_3_,c_2_,c_3_)) exhibited a microstructure predominantly composed of eutectic structures with supplementary hypereutectic constituents arranged in a lamellar-intergranular distribution. At 2000 W, the middle and upper regions of the coating contained white lamellar reinforcing phases and black vertical/round reinforcing phases, respectively. When the power increased to 2250 W, white lamellar reinforcing phases were observed in both the middle and upper regions, with an increase in both the area fraction and grain size of these phases. As the laser power further increased to 2500 W, the area fraction of reinforcing phases in the middle region continued to expand, while their grain size decreased. Notably, black elliptical reinforcing phases reappeared in the upper region of the coating.

With the increase in laser power, the grain morphology transitioned from an initial polygonal shape to a more regular elliptical form, accompanied by a gradual enlargement of grain area and a shift in distribution from isolated dot-like patterns to continuous lamellar structures. Additionally, the originally elongated black grains tended to adopt elliptical morphologies as the power increased. These observations indicated that the thermal effects and rapid cooling under high laser power exerted a direct influence on grain morphology and distribution. The elevated laser power introduced higher thermal input, which promoted more thorough elemental fusion within the alloy and facilitated the formation of new phases. Simultaneously, it accelerated grain boundary migration rates and contributed to grain refinement.

During laser cladding, the CoCrFeNiMo HEA powder underwent rapid heating and cooling, where Mo atoms acted as solute atoms during solidification, leading to their redistribution within the FCC structure. Previous studies [25,39] demonstrated that the addition of Mo resulted in an increase in FCC lattice parameters. However, when the Mo content exceeded a critical threshold, stable FeCr-type σ phases formed in the FCC matrix. With a further increase in Mo content, FeMo-type σ phases emerged. The formation of these phases consumed a significant amount of Mo atoms, reduced the solubility of Mo in the FCC structure, and released lattice distortion energy, thereby decreasing the average lattice constant of the FCC phase. In this study, FeCr-type σ phases were referred to as σ_a_ phases, while FeMo-type σ phases were designated as σ_b_ phases.

Mo incorporation not only substantially enhanced lattice distortion but also impeded conventional atomic diffusion pathways. This dual effect effectively suppressed grain growth through two competitive mechanisms: the increased lattice distortion energy surpassed the grain boundary energy, creating an energy barrier for grain boundary migration; and the diffusion-limiting effect disrupted typical grain coarsening processes. These synergistic interactions resulted in a stabilized fine-grained microstructure. In this study, large grains formed by the σ phase were present at the bottom of the coating. As the laser power increased, the dilution rate of the coating increased, causing the σ_a_ phase at the bottom to transform into the σ_b_ phase. In the upper and middle parts of the coating, due to the different precipitation temperatures of the σ_b_ and σ_a_ phases during the solidification process, different eutectic and hypereutectic structures were formed.

A previous study [40] demonstrated that during the solidification process, the Mo content in the liquid phase exhibited a significant decrease compared to Cr, indicating the initial precipitation of the Mo-rich σ_b_ phase. As the temperature decreased, the FCC and σ phases co-precipitated. However, the limited content and short precipitation duration of the σ_b_ phase during early solidification stages hindered its growth. In the latter stage of solidification, the Cr content in the residual liquid phase decreased substantially, leading to the predominant formation of the σ_a_ phase. The progressive increase in σ-phase content induced the pronounced coarsening of interdendritic (ID) regions, ultimately resulting in the development of large-area network-like and blocky σ-phase configurations within the coating. In this study, the rapid solidification of the high-energy laser-induced melt pool led to the sequential precipitation and interleaved arrangement of the FCC and σ phases, forming a distinctive dual-phase architecture characterized by eutectic–hypereutectic hybrid structures.

**Figure 7:**
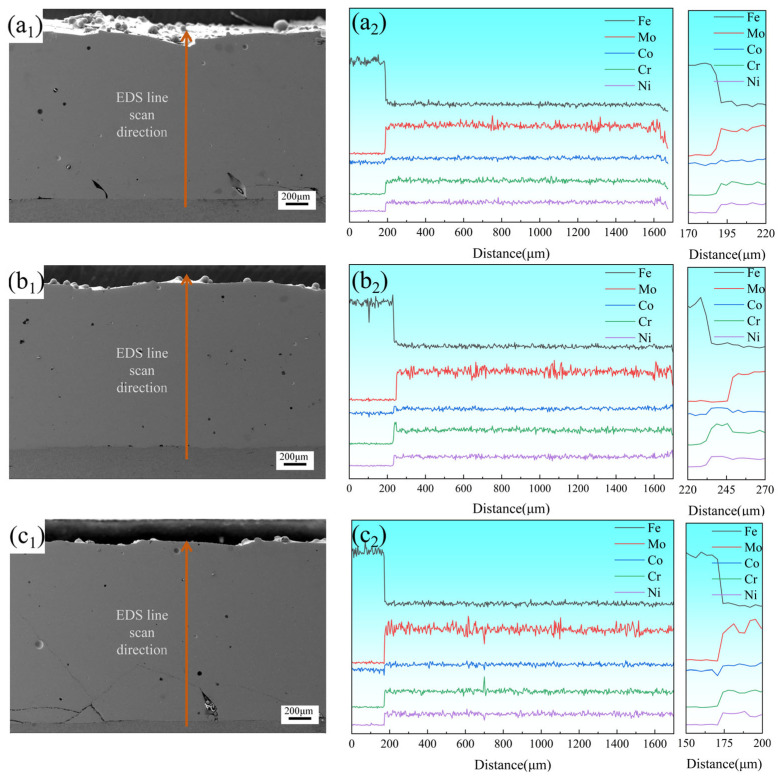
Overall morphology of CoCrFeNiMo HEA coatings and EDS element scans at corresponding positions: (**a_1_**,**a_2_**) 2000 W; (**b_1_**,**b_2_**) 2250 W; (**c_1_**,**c_2_**) 2500 W.

Figure 7(a_1_,b_1_,c_1_) present the overall morphologies of coatings prepared at 2000 W, 2250 W, and 2500 W, respectively, with a_1_, b_1_, and c_1_ displaying their corresponding EDS line-scanning profiles and magnified views. The coating fabricated at 2250 W exhibited the fewest defects, containing only minimal pores. EDS line-scanning results revealed significant compositional fluctuations in Mo compared to other elements, further confirming the interleaved arrangement of Mo-depleted FCC phases and Mo-enriched σ phases within the coating. The magnified EDS profiles demonstrated that a distinct diffusion transition zone formed at the coating–substrate interface. The significant elemental fluctuations in this zone indicated that when the laser power was 2250 W, the diffusion zone reached its maximum length of 30 μm. At this power level, the coating demonstrated both low dilution rates and strong metallurgical bonding, effectively maintaining the designed compositional integrity of the coating. Pronounced elemental fluctuations in this region indicated robust metallurgical bonding with low dilution rates, effectively preserving the designed compositional integrity of the coating.

**Figure 8:**
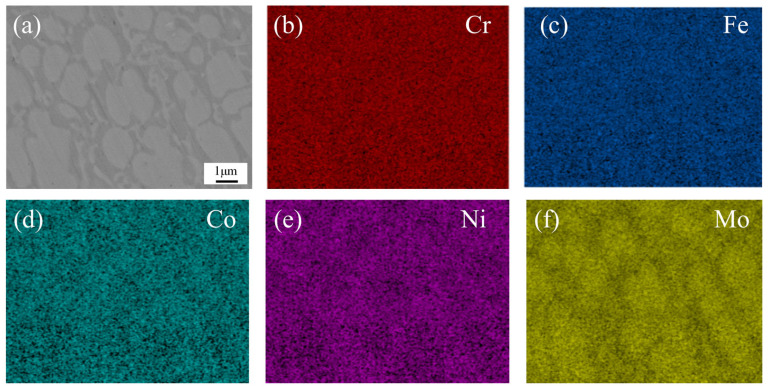
EDS at CoCrFeNiMo HEA coating with 1500 W power SEM morphology: (**a**) SEM morphology; (**b**) Cr; (**c**) Fe; (**d**) Co; (**e**) Ni; (**f**) Mo.

To further analyze the microstructural characteristics of the CoCrFeNiMo HEA coating, EDS mapping was performed on the middle region of the coating under 2250 W laser power. Figure 8 shows the results of the EDS mapping scan, where the enrichment of Mo elements in the white grains was clearly observed, while the gray region was noticeably deficient in Mo. Based on these observations, we inferred that the white grains corresponded to the σ phase, while the gray boundaries were identified as the FCC phase. The σ phase, known for its high hardness and corrosion resistance, was regarded as a strengthening phase in the alloy [41]. Simultaneously, the FCC phase exhibited excellent plasticity and toughness.

### 3.2. Mechanical Properties

**Figure 9:**
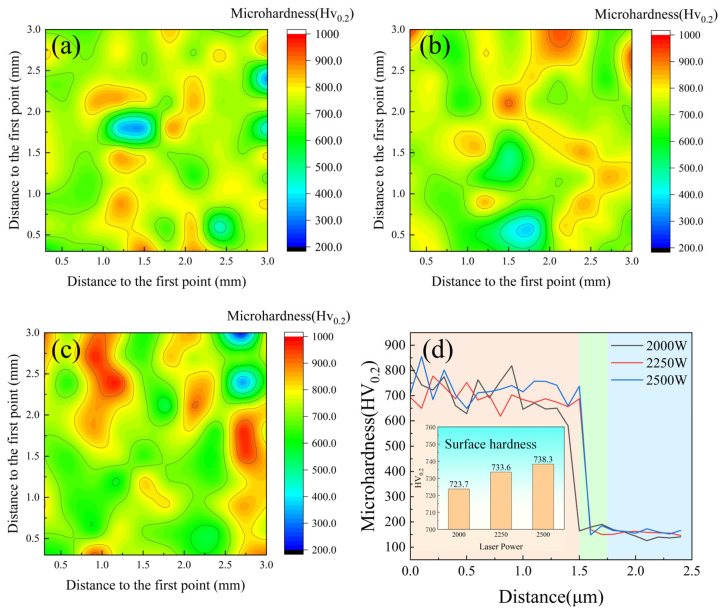
The hardness of CoCrFeNiMo HEA coating prepared by different laser powers: (**a**) 2000 W; (**b**) 2250 W; (**c**) 2500 W; (**d**) hardness of section.

As illustrated in Figure 9, the surface hardness analysis revealed that the overall trend of microhardness showed small variation across different conditions. As shown in Figure 9d, the Vickers hardness values measured 727.3, 733.6, and 738.3 HV_0.2_ for coatings produced at 2000 W, 2250 W, and 2500 W, respectively. These results demonstrate a gradual increase in coating hardness with elevated laser power. The enhanced hardness of the CoCrFeNiMo HEA primarily originates from three strengthening mechanisms: solid solution strengthening, grain refinement strengthening, and secondary phase strengthening. Particularly, the secondary phase strengthening is predominantly associated with the σ phase, which exhibits intrinsically high hardness and effectively impedes dislocation motion within the crystal lattice. Furthermore, the eutectic regions in the alloy possess a characteristic lamellar structure that can accommodate high dislocation densities, thereby significantly contributing to the overall hardness enhancement of the alloy.

In the CoCrFeNiMo HEA coating, the microhardness distribution at 2000 W exhibited numerous low-hardness regions, indicating significant hardness inhomogeneity. When the laser power increased to 2250 W, the low-hardness regions decreased, resulting in improved hardness uniformity. However, at 2500 W, the coating demonstrated the most pronounced hardness inhomogeneity, with the maximum difference between the lowest and highest hardness values. At a power of 2000 W, due to insufficient heat input, the lifetime of the molten pool was not long enough to allow the Mo element to diffuse uniformly, resulting in a small lattice distortion effect in the FCC phase. This led to the presence of many low-hardness regions in the coating, causing hardness inhomogeneity. In contrast, at a power of 2250 W, the lattice distortion of the FCC phase was sufficient, and the distribution of the σ phase was uniform, forming a dense network-like eutectic structure, which improved the overall hardness uniformity. However, when the laser power was further increased to 2500 W, the lifetime of the molten pool increased, allowing enough time for the σ phase to precipitate during the solidification process. The extensive precipitation of the σ phase reduced the solubility of Mo in the FCC phase, resulting in a lower degree of lattice distortion and a decrease in hardness, which created a significant hardness difference throughout the entire coating.

Figure 9d demonstrates the cross-sectional hardness characteristics of the CoCrFeNiMo HEA coating under different laser power conditions, from left to right are the coating, heat-affected zone, and substrate, distinguished by different color zones, with an average coating thickness of 1.5 mm. The coating exhibited relatively uniform hardness distribution throughout the cross-section, although minor fluctuations were observed, which could be attributed to factors such as melt pool dynamics, cooling rates, and interfacial interactions with the substrate. Furthermore, the heat-affected zone displayed a comparatively limited area, indicating effective control of thermal input during the laser cladding process that minimized thermal damage and microstructural alterations in the substrate. During laser cladding, the high-energy beam and rapid non-equilibrium cooling refined the grain structure of the CoCrFeNiMo HEA coating, while σ-phase precipitation enhanced hardness to about 730 HV_0.2_ because of the structure of the TCP phase. Benefiting from the intrinsic characteristics of the HEA system and precise laser processing parameters, the coating hardness reached 3–4 times that of the substrate material.

**Figure 10:**
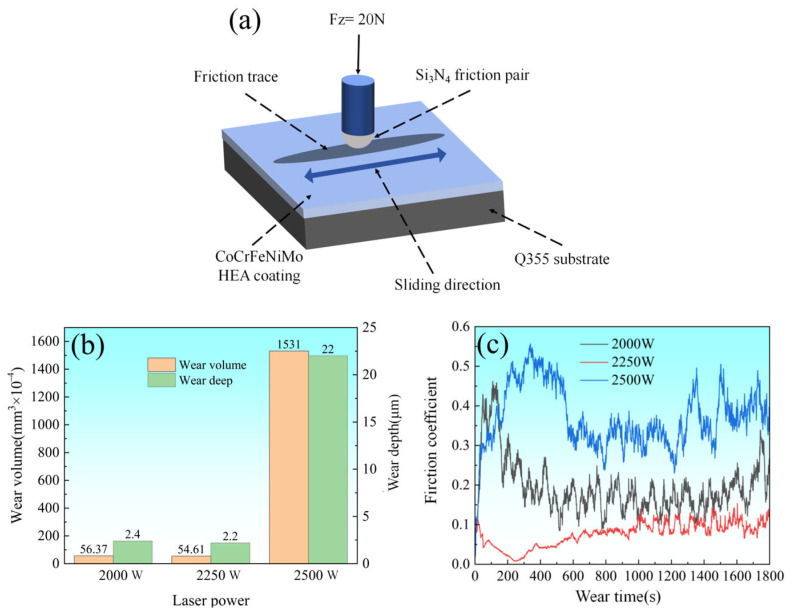
Schematic diagram of friction and wear experiment, friction coefficient, wear volume, and wear depth of CoCrFeNiMo HEA coatings with different laser power settings. (**a**) Schematic diagram of friction and wear experiment; (**b**) wear volume and wear depth; (**c**) coefficient of friction.

Figure 10a illustrates the schematic of the reciprocating wear test configuration, where a Si_3_N_4_ ceramic ball with a radius of 2 mm was employed as the sliding counterpart. The test was conducted under a normal load of 20 N at a frequency of 10 Hz for 30 min. As shown in Figure 10b, the wear volume and wear depth exhibited distinct laser power dependencies. The coatings prepared at 2000 W and 2200 W demonstrated comparable wear volumes, whereas the 2500 W sample showed significantly higher material loss.

Figure 10c presents the coefficient of friction (COF) performance of CoCrFeNiMo HEA coatings prepared under different laser power conditions. The plot reveals two distinct stages during the friction and wear process: the running-in period and the steady-state wear period. During the running-in period, the COF exhibited significant fluctuations, which stabilized after approximately 200 s, marking the transition to the steady-state wear period. However, even during the steady-state wear period, the COF demonstrated notable oscillations, indicating substantial inhomogeneity in the tribological properties of the coatings. These fluctuations in friction coefficient were primarily attributed to the asperity interactions between the coating and sliding counterpart, where the micro-protrusions generated non-uniform pressure distribution across the contact surface, consequently inducing variations in frictional behavior. Furthermore, the heterogeneous distribution of phase structures (FCC and σ phases) within the coating significantly contributed to friction inhomogeneity. The FCC phase, typically demonstrating superior ductility, provided enhanced deformation accommodation, while the hard σ phase, with its characteristic high hardness, offered exceptional wear resistance through load-bearing capability enhancement.

The CoCrFeNiMo HEA coatings at different laser power settings show significant differences in friction coefficients during the steady wear stage. Specifically, the friction coefficients of the coatings at 2000 W, 2250 W, and 2500 W power settings were 0.172, 0.084, and 0.371, respectively. It is clear that the coatings at 2250 W power have the lowest friction coefficients, whereas the highest friction coefficients are found for the coatings at the 2500 W setting. These variations are mainly attributed to the differences in heat input due to different laser powers, which, in turn, affects the content and ratio of FCC, σ_a_ phase, and σ_b_ phase in the coatings. The σ phase, especially as the TCP phase, effectively resists friction and prevents crack generation and grain detachment due to its high hardness. The σ_a_ and σ_b_ phases with different size ratios and their distributions also serve as a second reinforcement. Comparatively speaking, the FCC phase, due to its higher toughness, can effectively prevent crack extension and coating delamination during frictional wear. It can be hypothesized that the insufficient heat input at a laser power of 2000 W may lead to the generation of less σ phase and, thus, a higher friction coefficient. While at 2500 W laser power, the distribution of Mo-rich σ phase in the coating was uneven, and the excessive heat input caused phase transformation in the Q355 substrate, leading to the formation of cracks at the coating interface. In contrast, at a laser power of 2250 W, the distribution and balance of the σ phase and FCC phase were better, which not only helped prevent crack formation but also effectively prevented the propagation of cracks after they formed. Therefore, by optimizing the distribution and proportion of phases, the 2250 W setting performed best in maintaining the integrity of the coating structure while providing excellent wear resistance.

**Figure 11:**
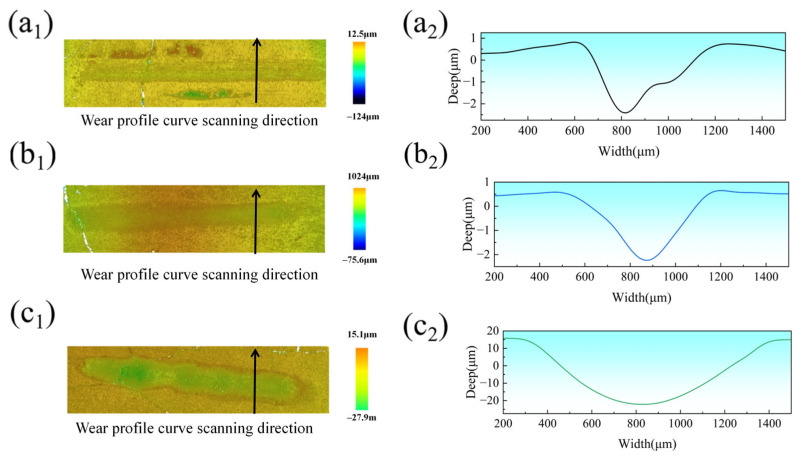
Three-dimensional wear morphology and wear profile of CoCrFeNiMo HEA coatings with different laser power settings: (**a_1_**,**a_2_**) 2000 W; (**b_1_**,**b_2_**) 2250 W; (**c_1_**,**c_2_**) 2500 W.

Figure 11(a_1_,a_2_,b_1_,b_2_,c_1_,c_2_) depict the 3D morphologies and wear profiles of the coatings after tribological testing at laser power levels of 2000 W, 2250 W, and 2500 W, respectively. The wear tracks exhibited elongated geometries, with V-shaped cross-sectional profiles for the 2000 W and 2250 W coatings showing relatively consistent depth from left to right. In contrast, the 2500 W coating displayed U-shaped wear cross-sections with pronounced depth fluctuations compared to the other parameters. Wear rates were calculated using Equation (2) [42]. The wear rates of the CoCrFeNiMoHEA coatings produced at 2000, 2250, and 2500 W were 4.691 × 10^−5^, 4.550 × 10^−5^, and 1.275 × 10^−3^ mm^3^N^−1^m^−1^, respectively.

(2) $$P=\frac{V}{LS}$$

where *P* represents the wear rate (mm^3^N^−1^m^−1^), *V* denotes the wear volume (mm^3^), *L* stands for the applied load (N), and *S* signifies the total reciprocating sliding distance (m).

**Figure 12:**
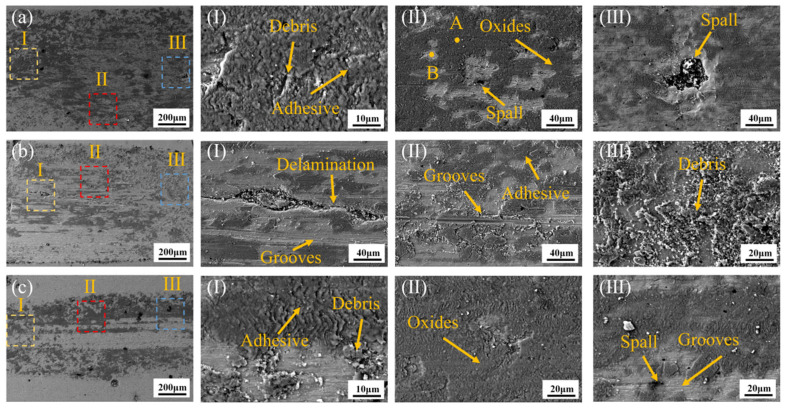
Wear morphologies of CoCrFeNiMo HEA coatings with different laser power settings: (**a**) 2000 W; (**b**) 2250 W; (**c**) 2500 W.

Figure 12 shows the wear morphology of the CoCrFeNiMo HEA coatings fabricated at different laser power levels after friction-wear tests. Figure 12a presents the wear morphology of the coating prepared at 2000 W laser power. In Region I, a large number of debris and adhesion marks are clearly visible, while in Region II, oxidation and grain spalling are observed. In Region III, larger grain spalling is present. EDS point scans were performed at points A and B in Region II. The results, shown in Table 2, indicate that the oxide formed on the coating is primarily Fe oxide, while Point B is rich in the Mo phase. During dry friction, a significant amount of heat was continuously generated, leading to the formation of an oxide film. The formation of the oxide film reduced the adhesion between the sliding surfaces, thereby effectively reducing the friction coefficient. As friction continued, the content of the oxide film gradually increased, resulting in the formation of adhesion marks. When the oxide film reached a certain level, the elements that form the oxide film were nearly depleted. As the oxide film cracked, it could no longer effectively protect the coating, and the hard phases began to peel off as a whole. The wear mechanism of the 2000 W coating is primarily oxidative wear.

Figure 12b illustrates the wear morphology of the coating prepared at 2250 W. Notably, compared to 2000 W, the content of the oxide film decreased. Grooves and delamination features were predominantly observed in Region I. This transition suggests that the 2250 W coating’s elevated hard-phase fraction (σ-phase) intensified brittle spallation phenomena while enhancing abrasive wear. The fragmented oxide layers were progressively comminuted into fine debris during sliding, as evidenced by SEM mapping showing discontinuous oxygen distribution. In summary, the wear mechanisms of the coating produced at 2250 W were primarily a combination of abrasive wear, oxidative wear, and adhesive wear.

Figure 12c illustrates the wear morphology of the coating prepared at 2500 W. As observed in Figure 12c, the formed oxides were predominantly concentrated along the flanks of the wear track, while the central region exhibited extensive grooving and spallation features. In the 2500 W coating, the oxide films failed to provide adequate protection, as evidenced by their discontinuous morphology and reduced adhesion strength. The detachment of hard σ-phase particles during sliding created abrasives that exacerbated subsurface cracking, resulting in significant material loss. The dominant wear mechanism transitioned to abrasive wear supplemented by localized oxidative wear.

**Figure 13:**
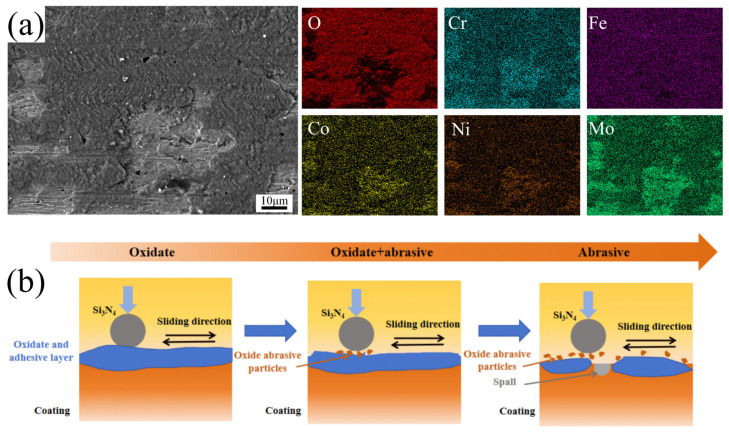
Wear morphologies and wear principle diagram of CoCrFeNMo coatings. (**a**) Wear morphologies; (**b**) wear principle diagram.

To further investigate the friction and wear mechanism of CoCrFeNiMo HEA coatings, an EDS mapping was conducted on the oxide layer formed after the coating’s friction and wear test. From Figure 13a, it is clearly observed that the black areas are oxide formations, primarily iron oxides, which some studies [43,44] suggest are γ-Fe_2_O_3_. The gray areas are enriched with Mo elements, which are presumed to be the σ-phase. During the process of resisting friction and wear, the ductile FCC phase both stabilizes the hard σ-phase to prevent large-scale grain loss and forms an oxide layer that reduces the wear rate. Meanwhile, the σ-phase, due to its extremely high hardness, limits the generation and propagation of cracks in the FCC phase.

Figure 13b presents a schematic diagram of the CoCrFeNiMo coating’s wear mechanism. During the sliding wear process, the high-speed friction between the Si_3_N_4_ ceramic ball and the HEA coating generated substantial frictional heating, gradually elevating the contact temperature. This thermal activation promoted the reaction between atmospheric oxygen and the elements in the HEA coating, resulting in the formation of a protective oxide layer. Wang et al. [45], through Raman spectroscopy, found that the oxides were mainly composed of Fe_3_O_4_ and Fe_2_O_3_, with the presence of MoO_3_, NiO, Cr_2_O_3_, and Co_3_O_4_ as well. Among these, Fe_3_O_4_ and Fe_2_O_3_ played a positive role in the tribological properties of the HEA coating by reducing the coefficient of friction and enhancing frictional stability. However, prolonged testing induced progressive stress accumulation within the oxide layer, triggering adhesive wear at the tribo-interface. As the experiment advanced, cyclic shear stresses fragmented the oxide film into abrasive debris. Deprived of oxide protection, the hard σ-phase particles underwent brittle spallation during subsequent sliding, generating micron-scale pits that accelerated material loss.

### 3.3. Corrosion Properties

**Figure 14:**
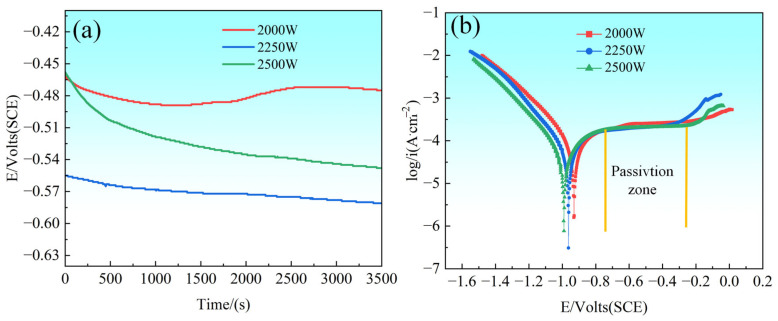
OCP and Tafel polarization curves of CoCrFeNiMo HEA coatings with different laser powers in 3.5 wt.% NaCl solution. (**a**) OCP; (**b**) Tafel polarization curves.

Figure 14 shows the results of open circuit potential (OCP) and polarization curve tests performed in 3.5 wt.% NaCl solution, which evaluated the corrosion behavior of CoCrFeNiMo HEA coatings prepared with different laser powers. As depicted in Figure 14a, the CoCrFeNiMo coatings with 2000 W showed a significant potential change process in the OCP tests, specifically a decrease followed by an increase and then a decrease again, which reflected the dynamic change of the coating surface passivation film; the initially formed passivation film may be locally dissolved under the action of corrosion medium and subsequently re-formed under electrochemical action and eventually destroyed again. In contrast, the CoCrFeNiMo HEA coatings of 2250 W and 2500 W show a relatively stable and slightly decreasing trend during the OCP test, which indicates that the passivation film formed on the surface of the coatings is able to provide a more sustained protective effect throughout the test period. In terms of specific values of corrosion potential, the CoCrFeNiMo coating at the 2000 W setting demonstrated the highest corrosion potential, indicating the lowest corrosion tendency.

The polarization curves shown in Figure 14b demonstrate the corrosion behavior of CoCrFeNiMo HEA coatings prepared at different laser powers in 3.5 wt.% NaCl solution. These curves reveal extremely stable passivation zones exhibited by the coatings at each power setting, and this stability indicates the formation of a durable passivation film on the surface of the coatings during polarization. The CoCrFeNiMo coatings show better stability during passivation, i.e., the current density remains almost constant during the increase in corrosion potential. In Sha’s study [46], Mo addition effectively refined the grain structure of HEAs and enhanced elemental distribution homogeneity, thereby reducing localized electrochemical potential differences.

The observation of pitting potentials reveals that the CoCrFeNiMo coating prepared at 2250 W laser power first reaches its pitting potential, indicating the earliest initiation of localized corrosion processes under this condition. In contrast, the 2500 W coating subsequently attains its pitting potential, while the 2000 W sample demonstrates the highest pitting potential, signifying its delayed onset of pitting corrosion at higher potentials and, consequently, superior resistance to localized corrosion in this specific test. The laser power not only governs the microstructure and phase composition of CoCrFeNiMo coatings but also significantly modulates their corrosion resistance, particularly in terms of passivation behavior and pitting resistance.

Table 3 presents the corrosion parameters of CoCrFeNiMo HEA coatings determined by the Tafel extrapolation method, including corrosion potential (*E*_corr_) and corrosion current density (*I*_corr_) under different laser power settings. The results demonstrate that both *E*_corr_ and *I*_corr_ values across the three laser power conditions exhibit minimal variation. Notably, the 2250 W coating displays the highest *E*_corr_ (−0.926 V vs. SCE) and the lowest *I*_corr_ (2.31 × 10^−4^ A·cm^−2^), confirming its optimal corrosion resistance among the tested conditions. Corrosion current density serves as a critical indicator for evaluating the corrosion rate of materials per unit time, where lower *I*_corr_ corresponds to reduced corrosion rates and enhanced durability. Consequently, the 2250 W parameter not only achieves superior corrosion potential but also demonstrates a 24% reduction in corrosion rate compared to the 2500 W counterpart.

**Figure 15:**
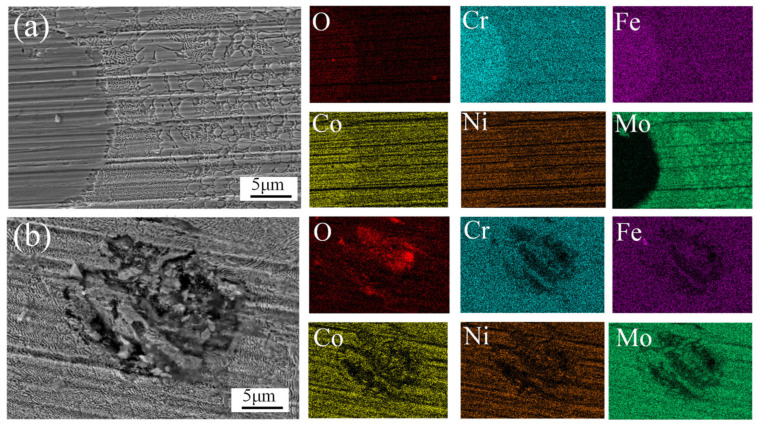
Typical morphologies of CoCrFeNiMo HEA after coating corrosion. (**a**) Grain spallation; (**b**) pitting corrosion pit.

To further investigate the corrosion mechanisms of the coating, two typical post-corrosion morphologies were selected, as shown in Figure 15. Figure 15a exhibits grain spallation, while Figure 15b displays a pitting corrosion pit. EDS analysis of the spalled grain in Figure 15a revealed a Cr-Fe-Co-O-enriched composition, with the surrounding matrix demonstrating Mo enrichment. As corrosion progressed, the selective dissolution of Cr, Ni, and Co occurred, forming pitting cavities as observed in Figure 15b. In the CrCoFeNiMo HEA coating, Mo acts as a chemically stable passivating element [47]. However, the electrochemical potential difference between the Mo-rich σ-phase and the Mo-depleted FCC matrix induced galvanic corrosion, accelerating localized degradation.

**Figure 16:**
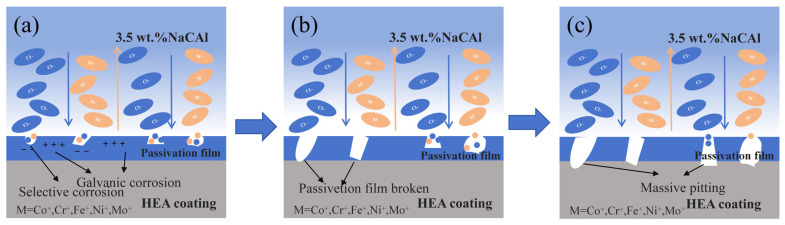
Electrochemical corrosion progression of the CoCrFeNiMo HEA coating. (**a**) First stage of corrosion; (**b**) second stage of corrosion; (**c**) third stage of corrosion.

Figure 16 illustrates the electrochemical corrosion progression of the CoCrFeNiMo HEA coating. The corrosion resistance of passivated alloys is intrinsically linked to the quality of their passive films. The passivation process generally proceeds through three sequential stages: 1. Anodic dissolution: The base alloy material dissolved via anodic reactions, releasing metal cations into the near-surface equiconcentration layer. 2. Cation diffusion: These cations subsequently diffused outward from the alloy surface, driven by concentration gradients and electrochemical potential. 3. Oxide nucleation and growth: Insoluble oxides/hydroxides (e.g., Cr_2_O_3_, MoO_3_) nucleated and coalesced on the surface, ultimately forming a continuous protective passive film.

In the CoCrFeNiMo HEA coating, the potential difference between the Mo-depleted FCC phase and the Mo-enriched σ phase created localized galvanic cells. This potential gradient accelerated the oxidation of Co/Fe in the FCC phase region, and the oxide film formed by Co/Fe could not effectively protect the coating, dissolving during the corrosion process. In addition, this potential difference facilitated the transfer of electrons at the phase boundaries, accelerating the selective dissolution of the coating.

The presence of Mo and Cr elements in the alloy, particularly their role in passive film formation, was of significant importance [48]. Cr was especially recognized as a crucial component for corrosion resistance due to its ability to form a stable Cr_2_O_3_ oxide layer. This oxide layer effectively prevented direct contact between corrosive media and the alloy matrix, thereby enhancing the overall corrosion resistance. However, the passive film underwent localized breakdown under pitting attack, exposing the underlying material and compromising its protective capability. The high corrosion potential facilitated the anodic dissolution of the matrix, potentially leading to the formation of metallic cations such as Mo^3+^. Although the hydrolysis of these cations could have induced severe acidification within the pit cavity, molybdenum oxides and their salt compounds formed and deposited within the pits, effectively inhibiting further pit propagation. Through this mechanism, the CoCrFeNiMo HEA coating demonstrated superior corrosion resistance in aggressive environments.

## 4. Conclusions

This study systematically investigates the microstructure evolution, tribological properties, and corrosion mechanisms of CoCrFeNiMo high-entropy alloy (HEA) coatings fabricated via coaxial powder-fed laser cladding under variable laser power conditions. The key findings and implications are summarized as follows:

(1) The CoCrFeNiMo coatings exhibited a dual-phase structure comprising FCC and σ phases. At 2250 W, optimized energy density promoted homogeneous dispersion of Fe-Cr-rich σ_a_ and Fe-Mo-rich σ_b_ phases within the FCC matrix, forming a refined eutectic–hypereutectic hybrid microstructure.
(2) The hardness of the CoCrFeNiMo HEA coatings increased with laser power, peaking at 738.3 HV_0.2_ at 2250 W. This improvement was attributed to solution strengthening and second-phase intensification. However, at 2000 W, the hardness decreased due to grain coarsening caused by the extended molten pool duration.
(3) At a laser power of 2250 W, the CoCrFeNiMo HEA coating exhibited the lowest coefficient of friction (COF = 0.084) and excellent wear resistance. This can be attributed to the oxide film formed during friction and wear, along with the eutectic structure composed of the FCC and σ phases, with the wear mechanism being oxidative wear.
(4) Electrochemical tests revealed that the CoCrFeNiMo HEA coatings achieved the best passivation film properties when at a laser power of 2250 W. The lowest corrosion current density (*I*_corr_ = 2.31 × 10^−4^ A/cm^2^) and the highest pitting potential are attributed to the formation of the passive film of Cr/Mo, as well as the reduction in the potential difference between the FCC (Mo-poor) and σ (Mo-rich) phases.

The CoCrFeNiMo HEA coating produced at 2250 W exhibits excellent mechanical and electrochemical performance, indicating strong potential for industrial application. The coaxial powder-fed laser cladding technique used in this work is compatible with industrial-scale processing due to its scalability, automation potential, and adaptability to complex geometries. Combined with the enhanced wear and corrosion resistance of the coating, this technique offers a promising solution for extending the service life of components in demanding environments, such as the marine or aerospace industries.

## Figures and Tables

**Table 1:** Chemical composition of Q355 steel (wt.%).

C	Si	Mn	P	S	Cr	Ni	Cu	Fe
≤0.24	≤0.55	≤1.60	≤0.035	≤0.035	≤0.30	≤0.30	≤0.40	Bal.

**Table 2:** Element content of wear morphologies (at.%).

Point	Co (at.%)	Cr (at.%)	Fe (at.%)	Ni (at.%)	Mo (at.%)	O (at.%)
A	4.88	7.09	16.39	5.67	4.63	61.34
B	18.00	18.84	18.54	17.31	18.45	8.87

**Table 3:** The Tafel fitting parameters of the polarization curves.

Sample	*E*_corr_ (mV/SCE)	*I*_corr_ (mA/cm^2^)
2000 W	−0.930	2.98 × 10^−4^
2250 W	−0.926	2.31 × 10^−4^
2500 W	−0.990	3.70 × 10^−4^

## Data Availability

The original contributions presented in this study are included in the article. Further inquiries can be directed to the corresponding authors.
